# An Exploratory Study of an fMRI Reward-Learning Paradigm in Developing Adolescents

**DOI:** 10.3390/children13050661

**Published:** 2026-05-09

**Authors:** Sarah Yale, Jeffrey Engelmann, Michelle Loman, DaJhnae Gambrell Sanders, Mohit Maheshwari, Theresa Mikhailov

**Affiliations:** 1 Division of Hospital Medicine, Department of Pediatrics, Medical College of Wisconsin, Milwaukee, WI 53226, USA; 2 Research Center, Rogers Behavioral Health, Oconomowoc, WI 53066, USA; jeff.engelmann@rogersbh.org; 3 Division of Child Neuropsychology, Department of Neurology and Pediatrics, Medical College of Wisconsin, Milwaukee, WI 53226, USA; 4 Department of Dermatology, Medical College of Wisconsin, Milwaukee, WI 53226, USA; dgambrellsanders@mcw.edu; 5 Department of Radiology, Medical College of Wisconsin, Milwaukee, WI 53226, USA; mmahesh@mcw.edu; 6 Division of Critical Care, Department of Pediatrics, Medical College of Wisconsin, Milwaukee, WI 53226, USA; tmikhail@mcw.edu

**Keywords:** vaping, electronic nicotine delivery systems, adolescents, functional magnetic resonance imaging

## Abstract

**Introduction:** Electronic nicotine delivery systems (ENDSs), also known as e-cigarettes or vapes, have shown popularity among the adolescent population. Compared to adults, less is known regarding the impacts of ENDS and nicotine on the adolescent brain. Adolescent research related to nicotine and other illicit substances can be difficult due to the requirement of parent/guardian consent, adolescent hesitancy for disclosure of product use, and the continually evolving vaping and nicotine products on the market. Despite these challenges, further research is needed to explore the impact of ENDS on the developing adolescent brain. The objective of the study was to evaluate reward sensitivity and cognitive flexibility in the adolescent population using functional magnetic resonance imaging (fMRI) through a probabilistic reversal learning task. **Methods:** This pilot study recruited participants aged 13–19 years old to complete fMRI testing. We specifically adapted a probabilistic reversal learning task that was previously used to measure reward sensitivity and cognitive flexibility in adults (including nicotine users). We were unable to recruit enough ENDS users to complete the planned analysis; therefore, we evaluated non-users as proof of concept for the use of the probabilistic reversal learning task in adolescents to support future research. Participants completed four blocks of a probabilistic reversal learning task, each lasting 6 min. During each block of the task, blood-oxygenation-level-dependent (BOLD) fMRI images were collected. The reward sensitivity and cognitive flexibility contrasts of parameter estimates were entered into a group analysis model. Due to the small sample size and exploratory nature of the study, we were interested in computing population-level estimates of brain activation that could be attributed to reward sensitivity (win-stay minus lose-stay trials) and cognitive flexibility (lose-shift trials minus lose-stay trials). **Results:** A total of twelve participants completed fMRI testing—ten non-users, one intermittent user, one regular user. Four of these participants (three non-users and one intermittent user) were excluded from the fMRI analysis due to excessive head movement and/or poor task performance. With the seven remaining non-users, we found no evidence of significant BOLD activation when strictly controlling the Type I error rate. Using a more liberal statistical threshold that did not control the Type I error rate, both contrasts resulted in suprathreshold clusters in occipital and posterior parietal regions, and the reward sensitivity contrast also resulted in suprathreshold clusters in the prefrontal cortex (bilateral middle occipital gyrus). **Discussion/Conclusions:** We did not find statistically significant BOLD activation, which is likely due to the small sample size. Suprathreshold clusters using the liberal statistical threshold may be feasible for use as regions of interest in future studies using this task. Notably, the prefrontal regions where the reward sensitivity contrast exceeded the liberal statistical threshold in our study were similar to those observed in previous studies of reward sensitivity in adults (including nicotine users) and adolescents. This pilot study explores the use of an fMRI reward-learning paradigm in the adolescent population, which can serve as a catalyst for future research related to nicotine use.

## 1. Introduction

Electronic nicotine delivery systems (ENDSs), also known as e-cigarettes or vapes, were introduced in the United States in 2006 as an adjunct to smoking cessation therapies, although they do not carry Food and Drug Administration approval for this indication [1]. With adolescent-targeted advertisement, youth-appealing flavoring, and ease of obtaining delivery systems, ENDS quickly reached the adolescent population. By 2018, 20.8% of high school students in the United States reported using ENDS in the past month. In early 2019, the Centers for Disease Control and Prevention declared that the ENDS epidemic had erased recent progress in preventing tobacco use among youth [2]. While ENDS use has declined since 2019, the 2024 National Youth Tobacco Survey revealed that use of any tobacco product still was reported by 10.1% of high school students (1.58 million) and 5.4% of middle school students (640,000 students) [3]. Additionally, new nicotine-containing products such as nicotine pouches have seen a concerning rise in US adolescents [4]. Depending on specific product features and individual user behavior, ENDS can deliver variable levels of nicotine [5], with the resultant potential for nicotine addiction in the developing adolescent brain. Compared to the adult population, less is known regarding the impacts of ENDS and nicotine on the adolescent brain. Prior work has demonstrated concern that nicotine exposure during developmental periods may be detrimental to executive function, reward-related circuitry, and learning and memory [6]. Research has also demonstrated that altered reward sensitivity is associated with smoking use in adolescents [7], but the neural mechanisms underlying these differences in reward sensitivity have not been extensively studied. With these knowledge gaps, there remains an ongoing need for further research, specifically in the developing adolescent brain.

Adolescent research related to nicotine and other illicit substances can be difficult due to the requirement of parent/guardian consent, adolescent hesitancy to disclose product use, and the continually evolving vaping and nicotine products on the market. Despite these challenges, this work is crucial to better understand the impact of ENDS on the developing adolescent brain. The objective of this pilot study was to evaluate two processes thought to be important in the development and maintenance of nicotine dependence, reward sensitivity and cognitive flexibility, in an adolescent population.

Reward sensitivity refers to a person’s behavioral and brain responses to rewards. Decreased reward sensitivity is an established phenotype of nicotine dependence in adults [8,9,10,11] but has not been as thoroughly studied in adolescents who use nicotine [12]. Prior research found that behavioral and brain indices of reward sensitivity decrease as a function of increasing nicotine dependence regardless of abstinence status [13]. Decreased reward sensitivity is thought to be important in the development and maintenance of substance use because most abused substances, including nicotine, directly activate the brain’s reward system, reinforcing substance use more strongly than alternative behaviors [14,15,16]. In children and adolescents, brain regions that are responsive to the delivery of reward, and thus thought to underlie reward sensitivity, include the dorsal and ventral striatum, anterior cingulate cortex, and medial prefrontal cortex [17,18,19,20,21]. We thus hypothesized that adolescents who use ENDS would show decreased responsiveness to reward delivery in these brain regions compared to non-users.

Cognitive flexibility refers to the ability to change responses as feedback changes. Cognitive flexibility is typically assessed using probabilistic reversal learning tasks, measuring brain responses to negative feedback delivered on trials after which an individual switches their response versus trials after which the original response persists. Increased activation in the anterior cingulate cortex, anterior insula, and dorsolateral prefrontal cortex, and decreased activation in the orbitofrontal cortex has been associated with response shifting after negative feedback [13,22,23]. One study found that brain activation associated with response shifting in these areas decreases during nicotine withdrawal, which is remedied by the smoking cessation medication varenicline [13]. As we were not manipulating abstinence status, we did not have a prior hypothesis about brain reactivity during cognitive flexibility differing between users and non-users, but instead planned to examine whether similar regions to those previously implicated in cognitive flexibility were activated in our sample.

## 2. Materials and Methods

### 2.1. Study Design

We developed this pilot study to evaluate the effects of nicotine on the developing adolescent brain through measures of reward sensitivity and cognitive flexibility. We initially designed the study to also measure cardiovascular, pulmonary, and neuropsychological functioning in adolescents who do not use ENDS or those who use ENDS occasionally or regularly. We were unable to recruit enough participants who use ENDS for the cardiovascular, pulmonary, and neuropsychological analyses, but were able to recruit a small sample of non-users for the reward sensitivity and cognitive flexibility assessment. We completed this assessment using fMRI, which is a reliable, valid, and non-invasive measure of human brain activity. Specifically, we adapted a probabilistic reversal learning task that was previously used to measure reward sensitivity and cognitive flexibility in adults [22,23], including nicotine users. As we were unable to recruit a sufficient number of ENDs users to complete the planned analysis, we instead present data exclusively from the non-users as proof of concept for use of the probabilistic reversal learning task in adolescents for future research. This will be important as nicotine products available to adolescents are ever-changing and difficult for researchers and policymakers to keep up with.

We conducted this pilot study at a quaternary academic children’s hospital in the Midwest region of the United States. The multi-disciplinary research team comprised individuals subspecializing in pediatric critical care, pediatric hospital medicine, pediatric neuropsychology, addiction neuroscience, pediatric pulmonology, pediatric cardiology, radiology, and adolescent medicine. This study was approved by our institutional review board.

### 2.2. Recruitment

We recruited participants aged 13–19 years old through hospital and clinic visits, as well as through partnership with a local school district. Recruitment information included a QR code for a registration link that the parent/guardian of prospective participants could complete electronically. Parents/guardians who responded were then contacted by a research coordinator to pre-screen the adolescent participant’s eligibility. The telephone pre-screening reviewed contact information, confirmation of participants and parent/guardian fluency in English, history of medical conditions that would be exclusionary, and use of ENDS products (both lifetime and recent use).

### 2.3. Eligibility

Exclusion criteria included a history of moderate to severe traumatic brain injury (TBI), brain tumor, stroke, genetic syndrome, and/or intellectual disability; contraindications for fMRI (i.e., metal in the body); pre-existing pulmonary condition; pre-existing cardiovascular condition; and use of any combustible products (i.e., cigarettes, cigarillos, joints) at least once over the past 30 days or more than 100 times ever.

### 2.4. Procedures

Eligible participants were then invited to attend an initial visit to the translational research unit located in the children’s hospital. During this visit, a member of the research team explained the goals and procedures of the study and obtained written assent from the participants as well as written informed consent from a parent/guardian. Adolescent participants then completed an electronic survey that included basic sociodemographic characteristics (date of birth, gender, race/ethnicity, language, address/zip code, handedness and educational information) and specific details about ENDS use to classify them as regular users, intermittent users, or non-users.

### 2.5. Participant Classification

Definitions of regular users, intermittent users, and non-users were based on those used by the Wisconsin Youth Risk Behavior survey [24]. Regular users reported vaping 20 or more days out of the last 30 days, intermittent users reported vaping at least once during the prior 30 days, and non-users reported no vaping at all over the prior 30 days and having tried vaping no more than 10 times in their lifetime. These categories aligned with methods often utilized within the literature to examine group differences [25]. Participants then provided a urine sample, which was tested for cotinine, a nicotine metabolite.

### 2.6. Assessments

**Figure 1:**
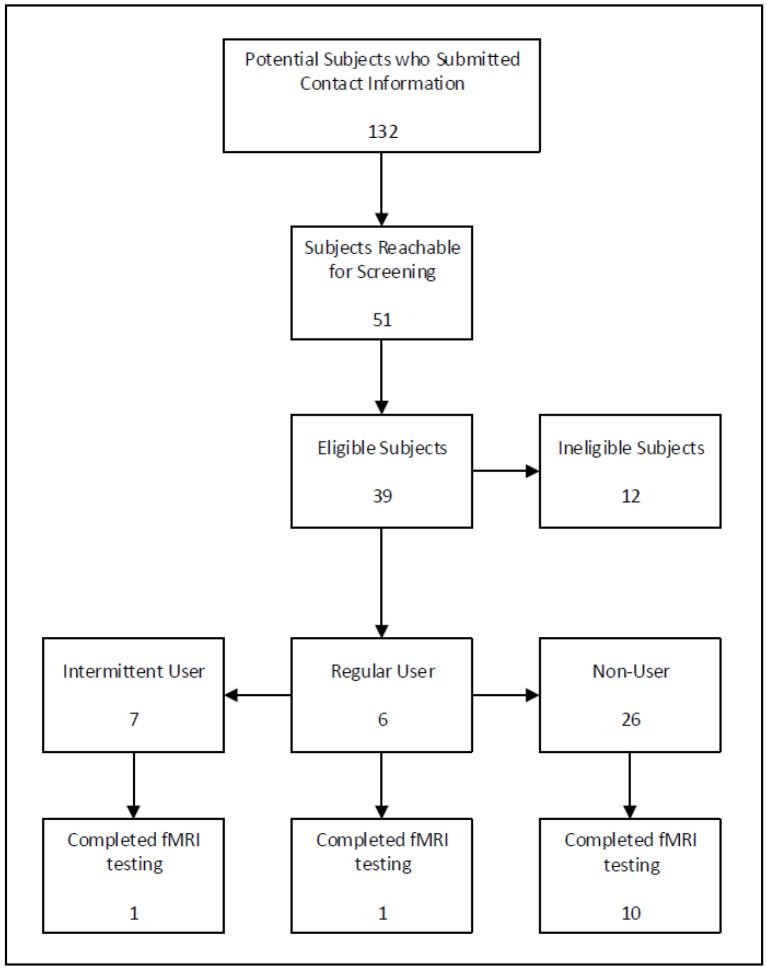
Recruitment and enrollment flowchart.

Participants who were non-users were only eligible to complete fMRI testing for reward sensitivity. Participants who were regular or intermittent users were eligible to complete fMRI testing as well as neuropsychological assessment. Every effort was made to complete all testing for a given participant within a two-week period, but all tests had to be completed within a 30-day period. A stipend for each part of the study completed was provided to participants. See Figure 1 for the recruitment and enrollment flowchart.

### 2.7. fMRI Data Collection

Participants completed a single neuroimaging session that lasted about one hour. Imaging was completed using a GE HealthCare (Waukesha, WI, USA) 3.0 Tesla Signa Premier magnetic resonance scanner with a 70 cm bore and gradients with a maximum strength of 80 mT/m and a maximum slew rate of 200 mT/m/s. At the start of the session, a T1-weighted anatomical image of the brain was obtained using a three-dimensional inversion-recovery gradient echo pulse sequence with the following parameters: matrix size = 276 × 276 × 224; flip angle = 8.0 degrees; inversion time TI = 900 ms; repetition time TR = 2411.77 ms; echo time TE = 2.72 ms. Magnetic field maps were constructed using two-dimensional spin echo echoplanar imaging acquisition sequences collected with opposite phase encoding directions (anterior-to-posterior and posterior-to-anterior). The parameters for these sequences were: acquisition matrix size = 108 × 108; reconstruction matrix size = 128 × 128; in-plane resolution 1.72 mm × 1.72 mm; 77 contiguous axial slices; slice thickness = 2.0 mm; flip angle = 90 degrees; TR = 8000 ms; TE = 65 ms. During each block of the probabilistic reversal learning task, blood-oxygenation-level-dependent (BOLD) fMRI images were collected using a two-dimensional, hyperband, gradient echo echoplanar imaging sequence with the following parameters: acquisition matrix size = 180 × 108; reconstruction matrix size = 128 × 128; in-plane resolution = 1.72 mm × 1.72 mm; 77 contiguous axial slices; slice thickness = 2.0 mm; hyperband acceleration factor = 3; flip angle = 90 degrees; TR = 2000 ms; TE = 25 ms. For each task block, 180 volumes were collected. The first two volumes from each run were discarded to allow T1 magnetization to reach a steady state.

### 2.8. Probabilistic Reversal Learning Task

Participants completed five blocks of a probabilistic reversal learning task, each lasting 6 min. This task has been previously used in fMRI studies of reward sensitivity and cognitive flexibility [13,22,23]. At the start of the session, participants were instructed that they would see two squares on the screen, one orange and one blue, and that one of the squares was “lucky” whereas the other was “unlucky”. They were told that if they chose the lucky square, they would most likely win points and only rarely lose points, whereas if they selected the unlucky square, they would most likely lose points and only rarely win points. They were told that their task was to get as many points as possible, as they would earn additional compensation (up to $10 United States dollars) based on how many points they earned. Participants were instructed to press a button on an MRI-compatible button box (Psychology Software Tools, Sharpsburg, PA, USA). After making their selection, they would be shown feedback that consisted of a happy face if they earned a point or a sad face if they lost a point. If they did not respond quickly enough, they were shown the feedback “too slow”. Finally, they were instructed that the lucky pattern frequently changes. The first block of the task was completed outside the scanner and used as a practice block. The remaining four blocks were completed inside the scanner while BOLD data were collected.

Each block consisted of 60 trials. The task was programmed and ran using E-Prime software (runtime version 3.0.3.60, Psychology Software Tools, Sharpsburg, PA, USA) and was presented via an MR-compatible stimulus display projected onto a mirror attached to the MRI receive coil. At the start of each trial, the two squares were shown (one on the left side of the screen and one on the right side of the screen) for 1500 ms. Participants had 1400 ms to make their choice (with the final 100 ms used to compute trial accuracy and prepare the feedback display). Feedback was displayed for 1000 ms, followed by an intertrial interval that consisted of a blank screen displayed for 1500, 3500, or 5500 ms. At the start of the procedure, one of the stimuli was randomly assigned as the lucky stimulus. For the lucky stimulus, 75% of responses were followed by positive feedback and 25% of responses were followed by negative feedback. After five consecutive correct responses (i.e., selecting the lucky stimulus) or twenty trials (whichever came first), the squares assigned as lucky and unlucky were reversed. The contingencies were reset at the start of each block. BOLD activity in response to the feedback display was the metric of interest. Thus, the task computer and scanner were time-locked according to when feedback was displayed, and the fMRI data were preprocessed and analyzed accordingly.

### 2.9. Behavioral Data Analysis

For each participant, the number of trials in each category and the mean reaction time for each trial category were computed. To determine whether there were differences in reaction time between the four main trial types (win-stay, win-shift, lose-stay, and lose-shift), a one-way within-subjects analysis of variance (ANOVA) was used with trial type as the within-subjects factor.

### 2.10. fMRI Preprocessing

Preprocessing of fMRI data was conducted using FreeSurfer 7.4.0, fMRI Software Library (FSL) version 6.0.7.13, and Analysis of Functional NeuroImages (AFNI) version 23.3.05.

T1-weighted images were preprocessed using FreeSurfer and FSL. First, skull stripping and intensity inhomogeneity correction were completed using FreeSurfer’s recon-all script using the -autorecon1 option [26,27]. Next, gray–white matter segmentation was completed using FSL’s FAST software version 6.0.7.13. Field maps were generated from the spin echo echoplanar images using FSL’s topup, brain extraction was performed using FSL’s bet2, and the images were co-registered to the T1-weighted anatomical images using FSL’s flirt.

For each run, the functional images were slice-time corrected using AFNI’s 3dtshift with Fourier interpolation. The first functional image from each time series was registered to the T1-weighted anatomical image using FSL’s flirt, with distortion correction applied using the field map images and boundary-based registration [28]. The time series was corrected for head motion using FSL’s mcflirt using a six-parameter rigid body transformation [29]. The T1-weighted anatomical image was registered to standard MNI152 space using FSL’s flirt using a 12-parameter affine transformation. The three transformations (head motion correction, functional-to-T1-weighted, and T1-weighted-to-MNI152) were combined to transform the time series data in MNI152 space with 2.0 mm^3^ resolution using sinc interpolation. Finally, the time series images in MNI152 space were blurred using AFNI’s 3dmerge -1blur_fwhm with a full-width at half-maximum of 5.0 mm and converted to units of percent signal change from the run’s time series mean for each voxel using AFNI’s 3dcalc.

### 2.11. fMRI Statistical Analysis

BOLD indices of reward sensitivity and cognitive flexibility were estimated for each participant using a voxelwise general linear model as implemented in AFNI’s 3dDeconvolve. Four regressors of interest were used to model BOLD responses to the presentation of feedback based on the feedback given (win or lose) and the participant’s response on the next trial (select the same stimulus, i.e., “stay”, or select the other stimulus, i.e., “shift”). We modeled win-stay trials, win-shift trials, lose-stay trials, and lose-shift trials. Previous studies did not explicitly model win-shift trials due to their low frequency; however, the number of win-shift trials in our sample varied considerably across participants (range = 3–51), so we modeled these trials. Trials in which participants responded too slowly and trials for which the next trial did not have a response (i.e., too slow trials and trials at the end of the block) were so infrequent that they were not modeled. The time courses of the feedback display on each of the four modeled trial types (win-stay, win-shift, lose-stay, and lose-shift) were modeled as square waves (1000 ms duration, matching the length of the feedback display) convolved with a canonical hemodynamic response function. Additional regressors were included to control for low-frequency scanner drift (third-order polynomial regressors for each run) and head motion (six head motion parameters for each run). Parameter estimates were computed using an autoregressive moving average model to control for temporal correlation in the fMRI data and restricted maximum likelihood estimation, as implemented in AFNI’s 3dREMLfit. We computed two contrasts of parameter estimates [13,23]. For reward sensitivity, we used win-stay trials minus lose-stay trials. For cognitive flexibility, we used lose-shift trials minus lose-stay trials.

We entered the reward sensitivity and cognitive flexibility contrasts of parameter estimates into a group analysis model. We did not have a sufficient number of participants to do comparative analysis between users and non-users as planned. Instead, we computed population-level estimates of brain activation that could be attributed to reward sensitivity (win-stay minus lose-stay trials) and cognitive flexibility (lose-shift trials minus lose-stay trials). This analysis was consistent with the exploratory nature of the study. We used a one-sample *t*-test for each contrast of parameter estimates, controlling for participant age, as implemented by AFNI’s 3dttest++. Group activation maps were corrected for Type I errors using a cluster size thresholding procedure as implemented by AFNI’s 3dClustSim [30]. For each of the two activation maps (reward sensitivity and cognitive flexibility), we controlled the Type I error rate at α = 0.05 using a single-voxel activation threshold of *p* < 0.001 and a minimum cluster size of 57 voxels, using AFNI’s Nearest Neighbor 1 (NN1) criterion for defining a cluster (faces of voxels must touch). In cases where there were no clusters that exceeded the activation threshold, we conducted further exploratory analyses with a liberal single-voxel activation threshold of *p* < 0.005 and a minimum cluster size of 40 voxels using AFNI’s NN1 criterion.

## 3. Results

A total of 132 potential subjects submitted contact information; after screening, there were 39 eligible subjects (Figure 1).

A total of twelve participants completed the fMRI testing—ten non-users, one intermittent user, and one regular user (Table 1). There were five female participants (42%) and seven male participants (58%). Participants ranged from age 13.1 to 17.9 years (median age 15.4 years). Four of these participants (three non-users and one intermittent user) were excluded from the fMRI analysis due to excessive head movement and/or poor task performance.

### Non-User Data

As the original aim could not be achieved due to recruitment constraints, this work was continued as an exploratory methodological exercise through analysis of data exclusively from the seven non-users.

Trial counts and reaction times for the seven non-users included in the fMRI analysis are presented in Table 2. There were no statistically significant differences in reaction time between the four main trial types (win-stay, win-shift, lose-stay, and lose-shift): *F*(3, 18) = 1.36, *p* = 0.29.

**Figure 2:**
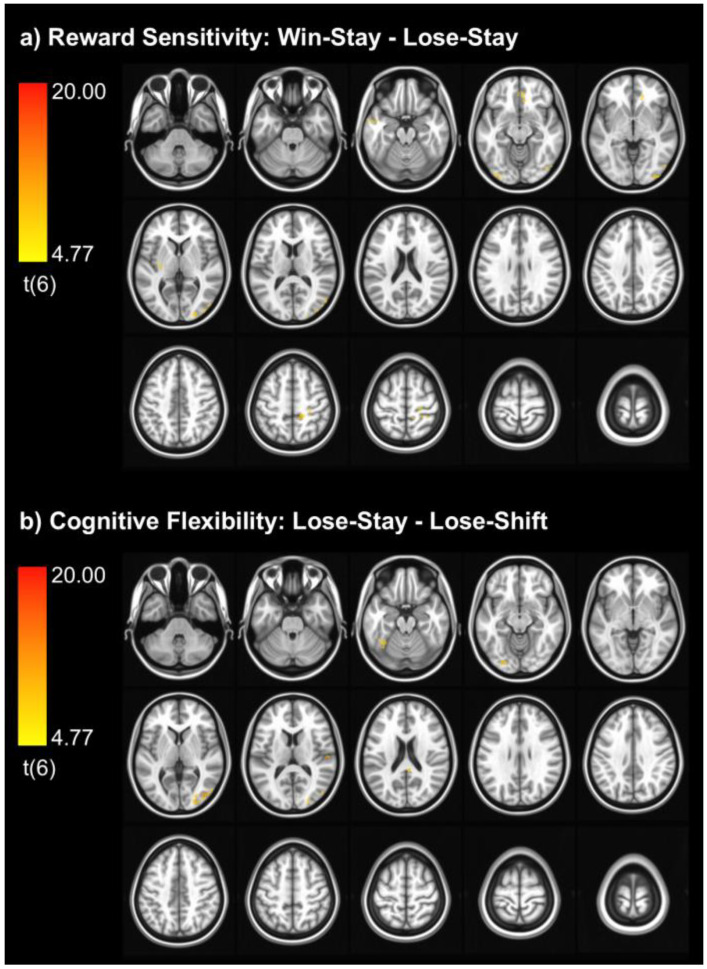
Suprathreshold BOLD clusters for the reward sensitivity (**a**) and cognitive flexibility (**b**) contrasts using the liberal statistical threshold: single-voxel *p* < 0.005 with a minimum cluster size of 40 voxels. This threshold did not control the Type I error rate. Axial slices are presented at intervals of 8 mm, starting at *z* = −36 mm in MNI space in the upper-left corner and moving across rows to *z* = 76 mm in the lower-right corner. The center slice is located at *z* = 20 mm.

At the stricter statistical threshold (Type I error rate α = 0.05, single-voxel *p* < 0.001, minimum cluster size 57 voxels), we found no evidence of BOLD activation for the reward sensitivity or cognitive flexibility contrasts. At the liberal threshold (single-voxel *p* < 0.005, minimum cluster size = 40 voxels), we found ten suprathreshold clusters for reward sensitivity and six suprathreshold clusters for cognitive flexibility (Table 3 and Figure 2). For reward sensitivity, suprathreshold clusters were located in occipital and posterior parietal regions (the left and right middle occipital gyrus, left lingual gyrus, and right middle occipital gyrus), near the right central sulcus (paracentral and postcentral gyri), bilaterally in the medial prefrontal cortex (middle orbital gyrus), and in the left putamen and middle temporal gyrus. For cognitive flexibility, suprathreshold clusters were located in posterior parietal and occipital regions (the right middle occipital gyrus, left fusiform gyrus, and left inferior occipital gyrus), and in the right superior temporal and posterior cingulate gyri.

## 4. Discussion

In the probabilistic reversal learning task, we did not find any activation clusters that exceeded the recommended, relatively strict threshold for controlling the Type I error rate [30]. At a lower statistical threshold that did not control the Type I error rate, we found suprathreshold clusters for both the reward sensitivity and cognitive flexibility tasks. These results should be interpreted cautiously, given the small sample size and liberal statistical threshold. They should not be thought of as clusters significantly activated by the task, but rather as potential regions of interest for future study using probabilistic reversal learning tasks in adolescents.

Both contrasts resulted in suprathreshold clusters in occipital and posterior parietal regions previously implicated in motivated attention during visual processing tasks [31,32,33], including responses to rewarding stimuli in adult cigarette smokers [34,35]. Given that our analysis modeled BOLD responses to feedback delivered during the task, these findings suggest that positive feedback was potentially more motivationally significant than negative feedback overall (reward sensitivity contrast), whereas negative feedback was more motivationally significant on trials in which participants switched their response to the other stimulus (cognitive flexibility contrast).

The reward sensitivity contrast also resulted in suprathreshold clusters in the middle occipital gyrus and putamen. These regions are thought to be part of a broad prefrontal-striatal network implicated in reward processing and substance use disorders [14,15]. Using this probabilistic reversal learning task, this frontal-striatal network was found to be active for the reward sensitivity contrast in both the general population of adults [22,23] and in cigarette smokers [13]. This network has also been implicated in cigarette craving in adolescents and young adults [36,37]. In smokers, the degree of activation in this network was inversely proportional to the level of nicotine dependence [13], which is consistent with previous studies in adults where blunted responses to rewards in this region predicted smoking lapse in a behavioral challenge task [38] and predicted smoking relapse during a quit attempt [35]. While limited by the small sample size, these findings explore a probabilistic reversal learning task as a possible measure of brain reward sensitivity in adolescents. As our analysis was limited to non-users, future research will be necessary to determine whether brain activation in these regions is modulated by nicotine use in adolescents.

In previous research using this task, the cognitive flexibility contrast found significant brain activation in prefrontal regions [13,22,39], an effect that was blunted by nicotine withdrawal and reversed by smoking cessation medications [13]. We did not find any suprathreshold clusters in this region for the cognitive flexibility task. This is most likely due to the small sample size or an insufficient number of trials in which participants switched responding to the other stimulus, but could also be due to developmental differences in neural substrates underlying cognitive flexibility [40].

Major limitations of this study include the small sample size of participants and the use of a liberal statistical threshold for the fMRI analysis. Recruitment issues included the need for parental consent in adolescents who likely did not want to disclose ENDS use to their parent/guardian, permanent orthodontia that would interfere with quality fMRI results, as well as polysubstance use other than nicotine. We hope that through dissemination of our learning related to recruitment barriers, we can aid other researchers looking to avoid similar pitfalls. A further limitation to sample size resulted from four participants who had poor task performance and/or excessive head movement in the fMRI scanner. For future studies, we recommend novel recruitment methods (such as exploring the use of recruitment through social media) as well as potential inclusion of polysubstance users so that the high exclusion rate in the adolescent population will not further limit the participant sample size.

We have experienced firsthand how research in the adolescent population related to nicotine or other illicit substances is complex, but this must not stop us from moving forward with this crucial work. Future work should include expansion of fMRI testing to a larger sample size of adolescents using nicotine-containing products (ENDS, pouches, etc.) and pairing this with further neuropsychological testing. Future research should focus on providing the necessary scientific evidence in support of advocacy for regulatory actions targeting nicotine-containing product use in adolescents.

## 5. Conclusions

This pilot study provides proof of concept for the use of the probabilistic reversal learning task that can serve as a foundation in the adolescent population, including for use with new and evolving nicotine substances. While there are significant limitations based on the small sample size, this pilot study demonstrates the feasibility of using an fMRI reward-learning paradigm, which can serve as a catalyst for future research related to nicotine use in the adolescent population.

## Figures and Tables

**Table 1:** Participant demographics, including user groups.

Age (Years)	Sex	User Group	fMRI Notes
14.99	Female	Intermittent	Excluded from fMRI analysis
17.9	Male	Regular	
16.59	Male	Non-User	
14.03	Male	Non-User	
15.72	Male	Non-User	
13.47	Male	Non-User	
13.13	Male	Non-User	Excluded from fMRI analysis
14.38	Male	Non-User	
17.05	Female	Non-User	
17.14	Male	Non-User	
13.7	Male	Non-User	Excluded from fMRI analysis
13.49	Female	Non-User	Excluded from fMRI analysis

Note: Those excluded from the fMRI analysis were removed due to poor task performance and/or excessive head movement.

**Table 2:** Behavioral data for non-users.

	Trial Count			Reaction Time (ms)		
Trial Type	Mean	SD	Range	Mean	SD	Range
Win-Stay	106.6	31.3	65–146	625.7	68.6	543–710
Win-Shift	23.6	20.4	3–51	586.0	111.7	466–741
Lose-Stay	41.4	21.3	16–79	628.7	93.1	523–801
Lose-Shift	59.6	12.6	39–81	626.6	84.2	526–719
Too Slow	2.6	1.5	1–5			
No response on next trial	6.3	1.4	5–8	641.3	117.7	476–850

Note: *n* = 7. Behavioral data collected from the four trial blocks during the fMRI scan. There were a total of 240 trials in the task across all four blocks. Mean trial counts do not sum to exactly 240 due to rounding error. Reaction times were not recorded for too slow trials.

**Table 3:** Suprathreshold clusters for the reward sensitivity and cognitive flexibility contrasts, controlling for age.

	Peak Voxel (mm)				
Region	x	y	z	Voxels	Maximum *t*
*Reward Sensitivity: Win-Stay—Lose-Stay*					
R Middle Occipital Gyrus	52	74	−10	262	14.57
R Paracentral Gyrus	14	36	50	88	17.48
L Lingual Gyrus	−36	88	−10	82	14.00
R Postcentral Gyrus	22	26	56	61	9.64
R Middle Occipital Gyrus	26	92	0	54	14.02
L Putamen	−28	8	0	50	9.55
R Middle Orbital Gyrus	12	−34	−8	47	10.57
L Middle Temporal Gyrus	−48	0	−16	44	9.51
L Middle Orbital Gyrus	−2	−40	−8	43	6.39
R Postcentral Gyrus	26	38	56	40	13.81
*Cognitive Flexibility: Lose-Shift—Lose-Stay*					
R Middle Occipital Gyrus	46	76	10	138	16.83
R Middle Occipital Gyrus	26	80	6	96	12.77
L Fusiform Gyrus	−38	60	−22	63	9.81
R Superior Temporal Gyrus	56	20	12	42	18.51
L Inferior Occipital Gyrus	−26	84	−12	41	9.41
R Posterior Cingulate Cortex	6	32	22	40	10.30

Note: *n* = 7 non-users. *R* = right hemisphere, *L* = left hemisphere. The *x*, *y*, and *z* coordinates are in MNI152 space and measured in mm. The statistical threshold used was a single-voxel of *p* < 0.005 with a minimum cluster size of 40 voxels using AFNI’s Nearest Neighbor 1 (NN1) method, i.e., faces of the voxels must touch. This threshold did not control the Type I error rate. Coordinates refer to the location of the peak voxel in the suprathreshold cluster. Voxel volume = 8 mm^3^ (2 mm × 2 mm × 2 mm); therefore, cluster volume is equal to the number of voxels in the cluster multiplied by eight.

## Data Availability

The original contributions presented in this study are included in the article. Further inquiries can be directed to the corresponding author.

## Abbreviations

The following abbreviations are used in this manuscript:

ENDS	Electronic nicotine delivery systems
fMRI	Functional magnetic resonance imaging
TBI	Traumatic brain injury
BOLD	Blood-oxygenation-level-dependent
